# Supplementary material for: Chromothripsis during telomere crisis is independent of NHEJ, and consistent with a replicative origin
Source: Genome Res. 2019 May;29(5):737–49. doi: 10.1101/gr.240705.118 (PMC6499312; doi:10.1101/gr.240705.118)
Supplement: Supplemental Material [file supp_gr.240705.118_Supplemental_file_1.zip › contigs/annotated_contigs/DB95/contig.3.DB95_length_709_mean_cov_8.98871650212.docx]

**DB95_length_709_mean_cov_8.98871650212**

```
TACTGAGTAGTGTTCCAATGAATGAATTTACTACAGTTGAGCATTTTCCTATGGATGGCTGCTAGGGCTGCTTCCTGTTTGGGATTACT
 >chr17:20992387-20992678 - E=3e-163
GTGAGTAAACCAGAAACGTACATTATAGTACAAGTCTTTTGGTGAAAATATATTTCATTCCTCTTGGTGCACACCAAAGGGTGGGACAC

TGGGTCAGAGAGTAGATGCATGTTTAGTTTTACAAGAAACTCCCAGGCCTAGGTTGCAGTGAACCAAGATCACACCACTCCACTCCAGC

CTGGGTGACAGAGTGAGACT|CCAT|TGTGCCCGGCCTTGGCTTTGTTTTCTGACTTCGTGCATTAACTGTTTCTTCTACACAGTAGTG
                     >chr17:20930824-20931014 - E=2e-99
TTTTCCTTGGAATGCTCATGGAGTTGGCAGGATATCCTTCAGAACAGCATTTTCAAGCGGTCCAAGCTAGAGACCTGTTTTAGGGAATA

GTTCATGTGAGCCTAGCAATTCCAGGTAAA|GATA|AATTTCTGAATTTTTTTGGTGTGTGTTATCTTGGAGATCATTTAGTTTCCTTG
                               >chr17:21051390-21051626 - E=4e-124
AAACTACTATTTTGAATTCTTTTTTTAAAAATTATTTTTTAGGGCTGGGCATGGTGGCTTCTCCCTGTAATCCCAGTGTTTTGGGAGGC

TTAGGTGGGAGGATTGCTTGAGCCCAGGAGTTTGACACCAGTCTGAGCAACATGGTGAGAACTTGTCTCTACAAAAAGTATAAAATAAA

T
```
